# Using linear algebra for protein structural comparison and classification

**DOI:** 10.1590/S1415-47572009000300032

**Published:** 2009-09-01

**Authors:** Janaína Gomide, Raquel Melo-Minardi, Marcos Augusto dos Santos, Goran Neshich, Wagner Meira, Júlio César Lopes, Marcelo Santoro

**Affiliations:** 1 Departamento de Ciência da Computação, Universidade Federal de Minas Gerais, Belo Horizonte, MGBrazil; 2 Departamento de Bioquímica e Imunologia, Universidade Federal de Minas Gerais, Belo Horizonte, MGBrazil; 3 Departamento de Química, Universidade Federal de Minas Gerais, Belo Horizonte, MGBrazil; 4 Laboratório de Bioinformática Computacional, Embrapa Informática Agropecuária, Campinas, SPBrazil

**Keywords:** protein classification, contact maps, linear algebra, singular value decomposition, latent semantic indexing

## Abstract

In this article, we describe a novel methodology to extract semantic characteristics from protein structures using linear algebra in order to compose structural signature vectors which may be used efficiently to compare and classify protein structures into fold families. These signatures are built from the pattern of hydrophobic intrachain interactions using Singular Value Decomposition (SVD) and Latent Semantic Indexing (LSI) techniques. Considering proteins as documents and contacts as terms, we have built a retrieval system which is able to find conserved contacts in samples of myoglobin fold family and to retrieve these proteins among proteins of varied folds with precision of up to 80%. The classifier is a web tool available at our laboratory website. Users can search for similar chains from a specific PDB, view and compare their contact maps and browse their structures using a JMol plug-in.

## Introduction

Proteins are an essential part of organisms and participate in every process within the cell. They catalyze biochemical reactions which are vital to metabolism, have structural and mechanical functions, play a crucial role in cell signaling and adhesion, and they are also involved in immune responses (Nelson and Cox, 2005; Branden and Tooze, 1999).

These macromolecules are organic compounds made of amino acids arranged in linear chains and joined together by peptide bonds between carboxyl and amino groups of adjacent amino acid residues. The sequence of amino acids in a protein is defined by the genetic code and is part of a set of 20 standard residues most commonly found in living creatures. An understanding of protein function is a crucial link in the development of new drugs, better crops and even in the development of synthetic compounds like biofuels.

Because of the rapid developments in genome sequencing technology and the inherently low throughput of the experimental procedures to elucidate protein function, the study of computational techniques which could help in protein function understanding is definitely critical. Many individual proteins of known sequences and structures present challenges to the understanding of their function. In particular, a number of genes responsible for diseases have been identified but their specific functions are unknown.

3D structures can aid in the assignment of function, motivating the challenge of structural genomics projects to make structural information available for novel uncharacterized proteins.

Structure-based identification of homologues often succeeds where sequence-alone-based methods fail, because in many cases evolution retains the folding pattern long after sequence similarity becomes undetectable.

Many methods of function prediction rely on identifying similarity in sequence and/or structure between a protein of unknown function and one or more well-characterized proteins. Alternative methods include inferring conservation patterns in members of a functionally uncharacterized family for which many sequences and structures are known (Whisstock and Lesk, 2003).

In an effort to contribute to advances in solving this problem, we propose a novel methodology to classify a huge dataset of proteins into protein families using conserved characteristics in structures of known function. We strongly believe that an important component of protein structural signatures is the pattern of chemical interactions between the chain residues in each family.

A contact map is a compact representation of the 3D conformation of a protein. It is defined as a symmetric square binary matrix in which the rows and columns are the residues of a protein chain and each point represents the interactions between the residues in the structure. Through a contact map, we can derive information about where protein α-helices and β-sheets are located in a given chain and also which parts of it are close to each other in 3D.

In the present work, we have built a matrix which represents a set of protein contact maps and, using linear algebra, we factorize that and compute an approximation with a specific rank which has fewer dimensions that the original dataset. We then use latent semantic indexing techniques to index and search the database. Up until now, we have only tested our methodology with hydrophobic interactions.

The growing size of protein databases, such as the PDB, provides strong motivation to apply this technique to the protein classification problem. Even though indexing such large datasets is a costly operation, it may be done incrementally and, once it is finished, queries can be answered efficiently.

Finally, using the similarity index used to answer the queries, we will show that the metric was able to retrieve myoglobins through a set of varied proteins with a precision of up to 80.64%.

## Material and Methods

###  Database selection

We use two data mart sets to apply our methodology. One is a sample dataset that is composed of five proteins of which three are myoglobins and the remaining two are proteins from different folds. We used the other database to evaluate the performance of our methodology. It is composed of fifty myoglobins and fifty proteins of varied folds selected from SCOP (Murzin *et al.*, 1995).

The selection of the proteins was made randomly from the proteins of the same species and we tried to select an approximate number of proteins of each species. The non-myoglobins were selected also randomly from proteins of similar size to myoglobins.

All of the biological data that came from the PDB and the hydrophobic contacts were calculated with 7 Å cutoff as proposed by Da Silveira *et al.*, 2009. The complete list of the proteins selected is displayed on the laboratory web site.

###  Building the contacts matrix

In order to use linear algebra for structural classification of proteins, we had to convert contact maps into vectors. In this work, from now on, the information about the 3D conformation of a protein in the contact map will be represented as a vector, which will be called a *contact vector*.

A contact map is a binary matrix. If the residues *x* and *y* make a hydrophobic interaction, so the position (*x*, *y*) of this matrix is 1, and is 0 otherwise. The contact vector is binary too and is a linearization of the contact map. The value of the element *i* of this vector is calculated by the Formula 1, where *b* is the number of residues of this protein.

*i* = (*x* - 1)(*b*) + (*y*)

**Formula 1.** Linearization of the contact maps.

The length of the contact vector is *b* x *b,* where *b* is the number of residues of the protein*.* As different proteins have different amounts of residues, the length of their contact vectors are also different. Consequently, it is necessary to make all of them the same size to make a matrix. In order to make all the contact vectors the same size, we take the size of the largest contact vector and normalize all the other contact vectors to this size.

After making all contact vectors the same size, we can group them all together and obtain a *n* x *m* matrix, where *n* is the number of proteins of the database and *m* is the size of the largest contact vector, that is *m = l*^*2*^, where *l* is the number of residues of the biggest protein of our database. This matrix is called *contact matrix*.

###  Indexing the proteins database through latent semantic indexing

The contact matrix is a large matrix and it is possible to take advantage of the implicit higher-order structure in the association of terms with documents in order to improve the detection of relevant documents on the basis of terms found in queries (Deerwester *et al.*, 1992).

Considering proteins as documents and contacts (from contact vectors) as terms, our goal is to build a retrieval system which should be able to find conserved characteristics in structures, represented by their hydrophobic interactions, and use them to classify a huge dataset of families. The linear algebra method that we used is called *Latent Semantic Indexing* (LSI) and it works by performing *Singular Value Decomposition* (SVD) of the contact matrix.

All things considered, from a large matrix of term-document association data, we construct a semantic space wherein intramolecular interactions and proteins that are closely associated and placed near one another. In other words, we can plot in 2D or 3D point representatives of proteins and contacts. SVD allows the arrangement of the space to reflect the major associative patterns in the data, and ignore the smaller, less important influences. For instance, contacts that did not actually appear in a protein, the atoms participating in its formation may still end up close to one another in the space, if it is consistent with the major patterns of association in the data.

In conclusion, position in space serves as a semantic index and retrieval can be achieved by using the contacts of a protein as a query to identify other proteins in the same space. Users retrieve the proteins in the neighborhood of the query (Deerwester *et al.*, 1992).

###  Defining the similarity metric for protein structure comparison

A common measure of similarity is the cosine between the query (*Q*) and the document vector (*D*) which is computed by the following:



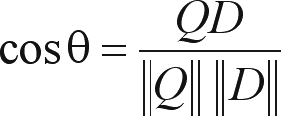


**Formula 2.** Cosine between the query (*Q*) and the document vector (*D*).

Typically, the *z* closest documents or all documents exceeding some cosine threshold are returned to the user. *z* is an integer number that represents the number of proteins that are similar to the query that you want to retrieve.

###  Classifying protein structures using the similarity metric

Using that similarity metric, we propose a protein structural classifier which retrieves proteins which are similar to a query based on the metric. In other words, each protein of the database has to be compared against the whole database. Finally we get, for each protein, all those ranked by their similarity (nearness) to it.

To determine the effectiveness of this retrieval system, we used a well-known statistical concept of *Confusion Matrix* and *Receiver Operating Characteristic* (ROC) curves, (Swets, 1988).

A confusion matrix (Provost and Kohavi, 1998) contains information about actual and predicted class assignments performed by a classifier and makes it possible to evaluate the precision of classification. This matrix gives the true-negative, true-positive, false-negative, and false-positive rates.

ROC curves are another way to examine the performance of classifiers. An ROC graph is a plot with the false-positive rate on the X-axis and the true-positive rate on the Y-axis. The false-positive rate is the number of negative instances predicted as positives divided by the number of negative instances. The true-positive rate is the number of positive instances predicted as positives divided by the number of positive instances.

In the ROC space, the point (0,1) is the perfect classifier: it classifies all positive cases

and negative cases correctly. It is (0,1) because the false-positive rate is 0 (none), and the true-positive rate is 1 (all). The point (0,0) represents a classifier that predicts all cases to be negative, while the point (1,1) corresponds to a classifier that predicts every case to be positive. Point (1,0) is the classifier that is incorrect for all classifications.

In many cases, a classifier has a parameter that can be adjusted to increase true-positives at the cost of increasing false-positives or decreasing false-positives at the cost of decreasing true-positives. Each parameter setting provides a (false-positive, true-positive) pair and a series of such pairs can be used to plot an ROC curve. In our algorithms, the parameter used is a threshold that we use to decide if a protein is or is not of a given family.

An ROC curve is independent of class distribution or error costs, and it encapsulates all information contained in the confusion matrix, since false-negatives are the complement of true-positives and true-negatives are the complement of false-positives. These curves provide a visual tool for examining the tradeoff between the ability of a classifier to correctly identify positive cases and the number of negative cases that are incorrectly classified.

Another interesting feature of these curves is that the area beneath them can be used as a measure of accuracy in many applications. Another way of comparing ROC points is by using a formula that equates accuracy with the Euclidean distance from the perfect classifier, point (0,1) on the graph.

###  Classifier calibration methodology

**Figure 1:**
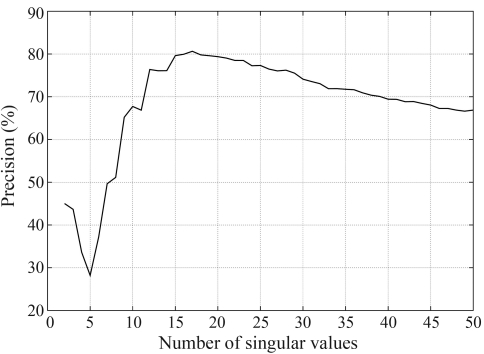
This figure shows the precision that we can obtain for each one of the possible singular values using linear algebra.

There are two important parameters to be adjusted in the system. The first one refers to the number of the singular values that is used to represent the data when we reduce its dimensions.

The number of singular values has to fit all the real structures in the data and try not to bring noise and redundant information to the representation. To discover this value, there is no simple rule but one way is to try all values and choose the one that best represents the data (Elden, 2006). Each parameter setting provides a (false-positive, true-positive) pair and a series of such pairs can be used to plot a ROC curve. We have conduced these experiments and plotted the ROC curves and found that, for myoglobins, the optimum parameter value was 17 dimensions, as shown in Figure 1.

The other parameter is related to the *z* number mentioned before, that is, how many proteins at the top of the rank to predict as positives. Once more, this value can be adjusted to increase true-positives at the cost of increasing false-positives or decreasing false-positives at the cost of decreasing true-positives.

###  Mathematical approach

In this section, we present the main concepts of LSI and SVD used to implement the proposed methodology.

Given an *mvs.n* matrix of documents and terms, *A*, and rank (*A*) = *r* ≤ *min*(*m,n*), the singular value decomposition of *A*, denoted by SVD(*A*), is defined as in Formula 3.

*A = D S T*^*t*^

**Formula 3.** The SVD of the matrix A is defined by this Formula. It decomposes matrix A into three others matrices, *D*, *S* and *T*^*t*^.

where *D*^*t*^*D = T*^*t*^*T* = *I*_*n*_ and *S* = *diag* (σ_1_, ..., σ_*n*_), σ_*i*_ > 0 for 1 ≤ *i* ≤ *r*, σ_*j*_ = 0 for *j* ≥ *r* + *1*. The *r* columns of the orthogonal matrices *D* and *T* define the orthonormal eigenvectors associated with the *r* nonzero eigenvalues of *AA*^*t*^ and *A*^*t*^*A*, respectively, (Deerwester *et al.*, 1992; Berry *et al.*, 1994; Elden 2006).

**Figure 2:**
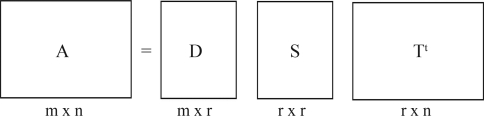
Schematic of the singular value decomposition of a rectangular document by term matrix. *A* is the matrix of document by terms, or proteins by their contact vectors; *D* has orthogonal, unit-length columns (*D*^*t*^*D* = *I*_*n*_); *T* has orthogonal, unit-length columns (*T*^*t*^*T* = *I*_*n*_); *m* is the number of rows of A, or the numbers of proteins; *n* is the number of columns of *A*; *n* is the number of columns of *A*, or the length of the contact vector; *r* is the rank of *A m* ≤ *min*(*m, n*).

Figure 2 presents a schematic of the singular value decomposition for a *mvs. n* of documents by terms.

The matrix *A* can be approximated in another matrix, *A*_*k*_, by modifying the three matrices that were factored above. From *S* matrix, the *k* largest singular values may be kept and the remaining smaller ones set to zero, in order to obtain the matrix *S*_*k*_. To obtain the new matrices *D*_*k*_ and *T*_*k*_ just keep the *k* first columns of the corresponding matrices *D* and *V.* It is important for the LSI method that the derived *A*_*k*_ matrix not reconstruct the original document term matrix *A*_*k*_ exactly (Berry *et al.*, 1994).

**Figure 3:**
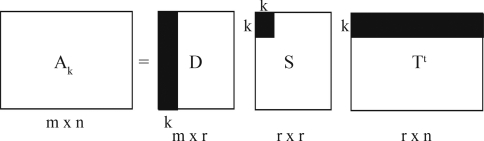
Mathematical representation of the matrix A_k_. This matrix is an approximation of the original document by term matrix using the *k* largest singular values and their corresponding singular vectors. *A* is the matrix of document by terms, or proteins by theirs contact vectors; *D* has orthogonal, unit-length columns (*D*^*t*^*D* = *I*_*n*_); *T* has orthogonal, unit-length columns (*T*^*t*^*T* = *I*_*n*_); *m* is the number of rows of *A*, or the numbers of proteins; *n* is the number of columns of *A*; *n* is the number of columns of *A*, or the length of the contact vector; *r* is the rank of *A* m *m* ≤ *min(m, n)*; *k* is the chosen number of dimensions in the reduced model (*k* ≤ *r*).

The SVD derives the latent semantic structure model from the orthogonal matrix, *S*, of singular values of *A.* These matrices reflect a breakdown of the original relationships into linearly-independent vectors or *factor values*. In some sense, the SVD can be viewed as a technique for deriving a set of uncorrelated indexing variables or factors, whereby each term and document is represented by a vector in *k*-space using elements of the left or right singular vectors. See Figure 3.

The choice of the value of *k* is a difficult job because it is related to dimension reduction. It has to be a value that fits all the real structures in the data and but small enough so that the noise and the redundant information do not fit in the new representation.

It is important to emphasize that for modeling the problem of structural classification of proteins through their intramolecular interaction we represent the matrix *A* as a matrix of documents by terms and not a matrix of terms by documents as in Deerwester *et al.* (1992), Berry *et al.* (1994), and Elden (2006). As said previously, the documents are the proteins and the terms are contact vectors representing the intramolecular interaction in the protein. The matrix *A* is done in this way just in order to make the processes of their construction more intuitive. This change in the meaning of rows and lines of matrix *A* doesn't modify the way that the singular value decomposition and the others steps are performed. To make the correspondence of this representation and the other one done in Deerwester *et al.* (1992), Berry *et al.* (1994), and Elden (2006) just replace the name of the matrices documents with terms and terms for documents.

###  Representing terms and documents in 2D or 3D

In order to visualize the distribution of proteins in space and understand it, we can plot the documents and the terms in the space.

Using the first column of *D*_*2*_ multiplied by the first singular value, σ_1_, for the x-coordinates and the second column of *D*_*2*_ multiplied by the second singular value, σ_2_ for the y-coordinates, the documents (protein) can be represented on the Cartesian plane. Similarly, the first column of *T*_2_ scaled by σ_1_ are the x-coordinates and the second column of *T*_2_ scale by σ_2_ are the y-coordinates for the terms (intramolecular contact). To represent it in 3D just do the same thing for z-coordinates (Berry *et al.*, 1994).

###  Queries

The three possible types of comparison between two terms:

Two documents

Two terms

Term and document

In this work, we analyzed only how close two documents are. To do that, the matrix *A*_*k*_ is used since it is presumed to represent the primary reliable patterns underlying the data in *A*. Consequently, the query, *q*, will be a document, represented by its contact vector.

This user's query must be represented as a vector in *k*-dimensional space and compared to documents as can be seen in Formula 4.



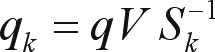


**Formula 4.** The user vectors are represented by this Formula, where *q* is simply the vector of contacts in the user's query, multiplied by the appropriate term weights.

###  Updating the database

Computing SVD of a large matrix is an expensive task computationally and may be impossible due to memory constraints (Berry *et al.*, 1994; Elden, 2006). If it's necessary to add new documents and/or terms in the matrix it's possible to do it without having to recompute the SVD.

This process is based in the existing latent semantic structure, the current *A*_*k*_. To add documents just calculate its *k*-space representation and then append it to the set of existing document vectors or columns of *D*_*k*_, as in Formula 5.



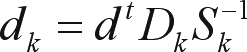


**Formula 5.** Cosine between the query (*Q*) and the document vector (*D*).

This process requires much less time and memory but can have deteriorating effects on the representation of the new terms and documents (Berry *et al.*, 1994).

###  Example

In this section, we present a full example of the application of the proposed methodology using five protein samples (3 myoglobins and 2 different structures). The analysis starts with the contact matrix, which is analyzed by singular value decomposition to derive the information that will be used. It will decompose the contact matrix into three other matrices of special form. These matrices have linearly independent components.

Many of these components are not significant and may be ignored, providing an approximate model with fewer dimensions. This makes it possible to represent the documents and the terms by *k* factor values, in other words, by the location of a vector in the *k* -space defined by the factors.

If the *k* value is low, just two or three factors, it is possible to view the distribution of proteins in space and understand it more clearly. However, when there are just a few factors, more factors have to be added to provide a better representation instead of visualizing them in 2D or 3D.

Due to the dimension reduction and the removal of redundant and/or noisy information by the method, it's possible for proteins, with somewhat different profiles of term usage, to be mapped into a similar vector and, more importantly, to find patterns of contacts that have been conserved in proteins of the same family.

To present an example of how queries are carried out with this example, we must represent a query as a *k*-dimensional factor space. The query will be a protein represented as a contact vector. After the query is transformed into a document in *k*-dimensional space, it can be compared against all others documents, and those with the highest cosines are the nearest vector and therefore the most similar (Deerwester *et al.*, 1992; Berry *et al.*, 1994).

**Figure 4:**
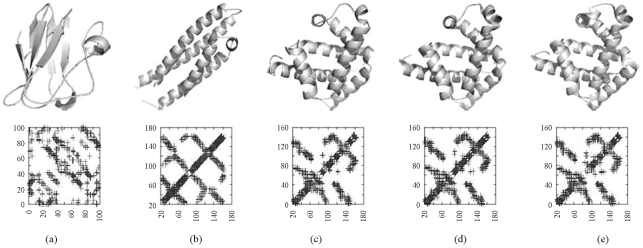
Different protein families and their contact maps. The protein 1ag6A (a) is a plastocyanin, 1b68A (b) is an apolipoprotein, 1emyA (c) is a myoglobin of the Asian elephant, 1l2kA (d) is a myoglobin of sperm whale and 1ycaA (e) is a pig myoglobin. This protein is the document of this sample dataset.

Table 1 shows a sample dataset. In this case, the documents are the proteins 1l2kA (a myoglobin of sperm whale), 1emyA (a myoglobin of Asian elephant), 1ycaA (a myoglobin of pig), 1ag6A (a plastocyanin) and 1b68A (an apolipoprotein). Figure 4 shows these proteins and their contact maps. The query will be the protein 1ycaA.

The entries in the term by document matrix are simply the contact vector of each protein normalized, as mentioned above. Such matrix could be used for the initial input of the SVD analysis. The query will be the protein 1ycaA (a pig myoglobin); in Figure 4 it's possible to see its structure and contact map.

**Figure 5:**
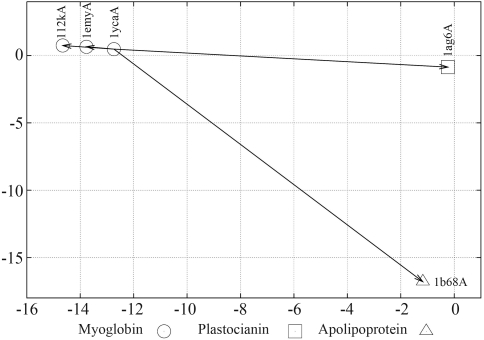
A two-dimensional plot of documents and the query from this sample dataset.

After the SVD computation it is possible to visualize the distribution of proteins in space, see Figure 5. The distribution of selected proteins in the 2D space places all myoglobins together, whereas plastocyanin and apolipoprotein are placed apart from all the other proteins. Thus, this method is capable of finding the pattern in the presentation of proteins using their contact maps by grouping the proteins from the same family, the myoglobins, and leaving apart the proteins that are different from the majority.

To determine which proteins are most similar to the query, we calculate the cosine of the vector the protein 1ycaA (the query) and the other proteins, see Table 2. Thus, the proteins most similar to 1ycaA are the proteins 1emyA and 1l2kA. These two proteins are myoglobins as in the query. And the less similar proteins are the other two that are not from this family.

## Results

In this section we analyze how the proposed approach performs in the retrieval of samples belonging to a given protein family from a set of proteins of varied topologies. We selected 50 samples of myoglobins and 50 samples from a variety of different families randomly constrained only by the chain size: we selected only chains which have between 100 and 200 residues as myoglobins have an average of 150 residues.

Our retrieval system receives a protein chain as a query and returns all the proteins ordered by the similarity to the query. In this way, if we use a myoglobin as a query, we expect that a perfect classifier returns all the other 49 samples of myoglobins at the top of the rank and the proteins of different topologies after that.

**Figure 6:**
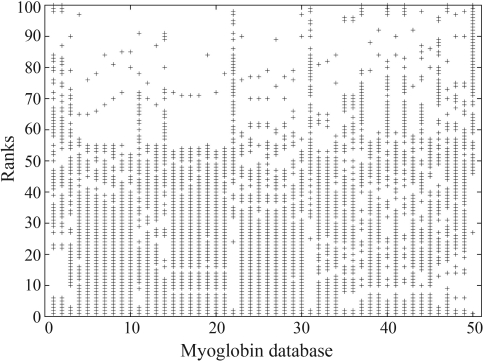
This figure shows all the ranks as lines and each column represents a chain of the database. A point in the picture means that the chain in that specific position of the rank is a myoglobin. The absence of point indicates that the chain in that position is not a myoglobin.

To access the precision of the system with the myoglobin dataset and have a general view of the behavior of the classifier, we plotted all the ranks in a single figure. In Figure 6, we show all the ranks as lines and each column represents a chain of the database. Notice that the figure presents 100 columns which are the 100 positions of each rank as the database has 100 elements and once you query the system, it returns the whole database ordered. We can also observe that we have 50 lines, each one representing the rank of a specific myoglobin as a query. In this experiment, we have used each of the 50 myoglobins as queries and compared them to the 100 chains of the database, which can be seen in the graph. A point in the picture means that the chain in that specific position of the rank is a myoglobin. The absence of point indicates that the chain in that position is not a myoglobin.

We can see that the majority of the myoglobins appear at the top of the ranking, and that the last 50 positions are mostly non-myoglobin proteins.

## Discussion

In this work, we describe a novel methodology to, using linear algebra, extract semantic characteristics from protein structures to compose structural signature vectors that we showed can be used to compare and classify protein structures in fold families efficiently.

We have computed the traditional contact maps using only hydrophobic contacts and a cut-off of 7Å. We then converted them into vector signatures and normalized all the vectors of the database to make them all the same size. This was done by straining the maps and moving pixels away.

Once all the contact vectors are the same size and are considered protein signatures, we used this data to index the protein database using latent semantic indexing. Considering proteins as documents and contacts as terms, we built a retrieval system which is able to find conserved characteristics in structures and have used it to classify proteins.

## Figures and Tables

**Table 1:** - Sample dataset consisting of five different proteins. The first three proteins are myoglobins and the remaining are proteins from different folds.

Documents (PDBId_Chain)	Terms (contact vector)
1L2K_A	000...000000...10...0...100000........000
1EMY_A	000...000000...10...0...100100........000
1YCA_A	000...000000...10...0...100000........000
1AG6_A	000...110001...00...1...100011........000
1B68_A	000...100000...00...1...000000........000

**Table 2:** - The cosine between the query (1YCA_A) vector and the other proteins. Note how the cosine between the myoglobins is very high and the cosine against the other two proteins is very low. The proteins set in italics are non-myoglobins.

Protein	Cosine	Rank
1YCA_A	1	1
1EMY_A	0.9999999978	2
1L2K_A	0.9999928293	3
*1AG6_A*	0.1691321587	4
*1B68_A*	0.0015279203	5
